# 2D speckle-tracking echocardiography assessment of left atrial and left ventricular mechanics: outcomes in patients with atrial fibrillation treated with hybrid ablation and left atrial appendage surgical closure

**DOI:** 10.3389/fbioe.2025.1538809

**Published:** 2025-03-12

**Authors:** Andrea Maria Paparella, Luigi Pannone, Gianni Pedrizzetti, Giacomo Talevi, Domenico Giovanni Della Rocca, Antonio Sorgente, Rani Kronenberger, Gaetano Paparella, Ingrid Overeinder, Gezim Bala, Alexandre Almorad, Erwin Ströker, Juan Sieira, Mark La Meir, Andrea Sarkozy, Pedro Brugada, Gian Battista Chierchia, Ali Gharaviri, Carlo De Asmundis

**Affiliations:** ^1^ Heart Rhythm Management Centre, Postgraduate Program in Cardiac Electrophysiology and Pacing, Universitair Ziekenhuis Brussel - Vrije Universiteit Brussel, European Reference Networks Guard-Heart, Brussels, Belgium; ^2^ Department of Engineering and Architecture, University of Trieste, Trieste, Italy; ^3^ Cardiac Surgery Department, Universitair Ziekenhuis Brussel - Vrije Universiteit Brussel, Brussels, Belgium

**Keywords:** atrial fibrillation, hybrid ablation, appendage closure, cardiac mechanics, hemodynamic forces

## Abstract

**Background and aims:**

Hybrid atrial fibrillation (AF) ablation is a therapeutic option in non-paroxysmal AF. Our study examines cardiac mechanics changes after hybrid AF ablation plus epicardial closure of left atrial appendage (LAA).

**Methods:**

All consecutive patients undergoing hybrid AF ablation at UZ Brussel were evaluated. They received pulmonary vein isolation (PVI), posterior wall isolation (LAPWI), and epicardial LAA closure. Left atrium (LA) and Left ventricle (LV) mechanics were analyzed, with the following measures obtained at baseline, post-ablation, and follow-up: 1) volumes (EDV, ESV); 2) ejection fraction (EF); 3) strain (ENDO GCS, ENDO GLS); 4) forces (LVLF, LVsysLF, LVim, LVs).

**Results:**

A total of 50 patients were included. At follow-up, LAEDV decreased from baseline [44.7 mL vs 53.8 mL, *P* = 0.025]. LA ENDO GCS and GLS increased post-ablation, with further GLS improvement at follow-up. LV ENDO GCS and LV ENDO GLS also rose post-ablation [-26.7% vs. −22.5%, *P* < 0.001] and [-20.57% vs. −16.6%, *P* < 0.001], respectively. LVEF increased post-ablation [54.6% vs 46.3%, *P* < 0.001]. There was an increase in all LV hemodynamic forces (HDFs) and in particular: LVLF and LVsysLF increased post-ablation [15.5% vs 10.4%, *P* < 0.001] and [21.5% vs 14.11%, *P* < 0.001], respectively. LVim also increased post-ablation [19.6% vs 12.8%, *P* < 0.001]. Finally, there was an increase in LVs post-ablation compared to baseline [10.6% vs 5.4%, *P* < 0.001].

**Conclusion:**

In patients undergoing hybrid AF ablation, there was a significant and persistent improvement in the mechanical and hemodynamic functions of both LA and LV.

## 1 Introduction

Since the description of pulmonary vein triggers, atrial fibrillation (AF) ablation has emerged as a therapeutic option for rhythm control. However, despite significant technological improvements, including novel energy sources, the success rate remains ≈70–80% for paroxysmal forms and 60%–70% for persistent forms at 1 year (Haïssaguerre et al., 1998). AF ablation involves the electrical isolation of the pulmonary veins (PVI), using various forms of energy such as radiofrequency, cryotherapy, laser energy, and electroporation (Mulder et al., 2022; Tzeis et al., 2024). Several studies have demonstrated that electrical PVI achieved with various energy sources significantly reduces the burden of AF in patients affected by this arrhythmia (Sorgente and Cappato, 2015). Transcatheter ablation has proven to be mainly effective in patients with paroxysmal AF (PAF), however, in patients with persistent AF (PersAF), the efficacy of medications and ablation is significantly reduced due to a more complex pathophysiological mechanism (Verma et al., 2015; Dan et al., 2018). In this context, thoracoscopic hybrid atrial ablation has emerged as an effective option for this challenging patient group (Marini et al., 2022a). This approach involves an epicardial ablation of the pulmonary veins (PVs) usually associated with the isolation of the posterior wall (LAPWI). The surgical part is also combined with an endocardial mapping and eventual ablation (hybrid ablation). Furthermore, the possibility to surgically close the left atrial appendage (LAA) in the same procedure represents a significant advantage. To date, in persistent and long-standing AF, the results of hybrid ablation in clinical experience (De Asmundis et al., 2017; Maesen et al., 2018; Pison et al., 2012; Pison et al., 2014) and randomized trials have been promising (Delurgio et al., 2020). However, there is limited data on the changes in cardiac mechanics and volumes after such approach, which may have significant clinical implications, especially considering: (1) the effect of the atrial lesion set and (2) the possible volumetric changes secondary to surgical LAA closure. The aim of this study is to evaluate the changes in cardiac mechanics after hybrid AF ablation. In particular, both a volumetric analysis and a functional analysis are aimed for both left ventricle (LV) and left atrium (LA).

## 2 Methods

### 2.1 Study population

All consecutive patients diagnosed with PersAF, who underwent hybrid AF ablation + epicardial LAA closure at UZ Brussel between 2010 and 2020 were screened. The following inclusion criteria were applied: (1) A confirmed diagnosis of AF according to current guidelines (Pannone et al., 2023); (2) Hybrid AF ablation procedure that included PVI + LAPWI and LAA closure. Patients were excluded if they had an intracavitary thrombus, were in a state of decompensated heart failure, had coronary artery disease, or moderate to severe valvular heart disease. It is important to note that during the selection of the patient sample, particular care was taken to exclude individuals whose echocardiographic image quality was deemed inadequate for analysis. This measure was taken to ensure the reliability and precision of the imaging analyses upon which this research is based.

### 2.2 Collection of echocardiographic images

During the echocardiographic imaging acquisition phase, particularly 2D echo images, the EchoPac (GE Healthcare, Chicago, USA) and Intellispace Cardiovascular Lab (Philips, Eindhoven, Netherlands) software were used at UZ Brussel. The images of patients included in the study were collected at three key time points: 1 week before ablation, 1 week after and after a median follow-up of 62.3months ±20.3. The echocardiographic images collecting phase was preceded by a careful verification process to ensure the availability and quality of images obtained through two-chamber, three-chamber, and four-chamber apical views, as these served as the fundamental reference for subsequent imaging analyses of the LV and the LA.

All acquired images were subsequently exported and saved in DICOM format, to prepare them for imaging analysis.

### 2.3 Echocardiographic imaging analysis of left atrial and left ventricular mechanics

2D speckle tracking echocardiography analysis of LA and LV mechanics was conducted using the software (QStrain; Medis Medical Imaging Systems B.V, Leiden, Netherlands).

#### 2.3.1 Left ventricle

The analysis of LV 2D strain was performed in all three apical views (LV four-chamber, two-chamber, and three-chamber). In the most suitable cardiac cycle, we manually traced the LV end-systolic borders, followed by the adjustment of the borders at end-diastole, throughout the entire cardiac cycle without altering the previously drawn contours. This approach allowed for the calculation of global endocardial circumferential strain (ENDO GCS), global endocardial longitudinal strain (ENDO GLS), end-systolic volume (ESV), and end-diastolic volume (EDV), as well as the measurement of left ventricular ejection fraction (LVEF) (Faganello et al., 2020; S1, Supplementary Methods).

**FIGURE 1:**
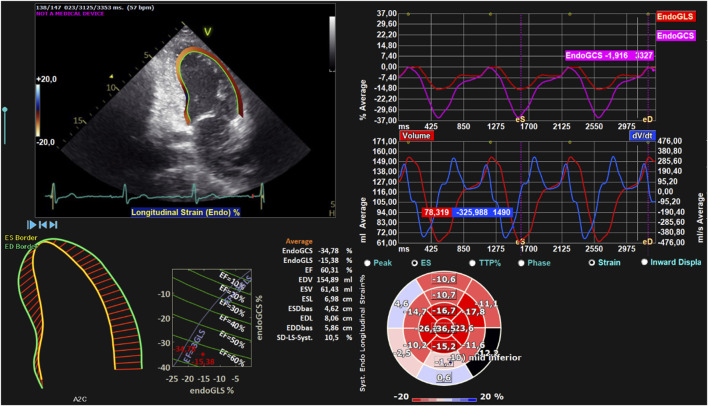
Left ventricular strain. Global analysis of a patient’s LV with endocardial contour and retrieval of ENDO GCS, ENDO GLS, EDV, ESV, and EF parameters. On the top left, endocardial contouring of the LV is shown. On the bottom left, LV end-diastolic and end-systolic contours, shown in green and yellow, respectively. On the top right, the strain trend throughout the entire heartbeat is displayed. On the bottom right, the volumetric trend (in red) and its temporal derivative (in blue) are shown, along with the strain bull’s-eye plot.

An example is presented of how, after LV endocardial border has been traced manually in the three apical views, a simulation of the contour deformation over an entire heartbeat was performed (Figure 1). In the echocardiographic image (top left), we can see a frame of the entire beat with the simulation of LV endocardial contour. At the bottom left, the contours at end-diastole (green) and end-systole (yellow) are respectively represented. At the top right, the graph shows the percentage trends of the ENDO GCS and ENDO GLS parameters, beneath which there is also the graph of the volume trend and the temporal derivative of the volume, all throughout the entire heartbeat. Finally, at the bottom centre, a series of parameters including those of our interest are displayed.

Once the parameters described above were obtained through contour tracing, left ventricular hemodynamic forces (HDFs) were calculated (Supplementary Figure S1). These measurements, in addition to providing detailed information about the pumping force and the dynamics of circulation, represent an innovative non-invasive measure of ventricular function (Arvidsson et al., 2022). The analysis of HDFs represents a promising approach for studying blood flow within the ventricular cavities through the exploration of intraventricular pressure gradients. Previous experimental studies have highlighted the importance of invasively measured cardiac pressure gradients in patients with heart failure. Subsequently, advances in cardiovascular imaging have enabled the non-invasive evaluation of pressure gradients during the progression and resolution of ventricular dysfunction and in the context of resonance therapy. The analysis of HDFs can amplify mechanical abnormalities, detecting them earlier than conventional analysis of ejection fraction and strain, and potentially predicting the model of cardiac remodelling. Changes in HDFs provide early signals of impaired cardiac physiology and can thus transform the existing paradigm of cardiac function analysis once implemented in routine clinical practice. Until recently, the investigation of HDFs was only possible with contrast-enhanced echocardiography and magnetic resonance imaging, limiting its widespread clinical application (Vallelonga et al., 2021). Therefore, HDFs analysis could represent an innovative tool useful in preventive cardiology, capable of detecting alterations in cardiac physiology in asymptomatic individuals and offering an opportunity for early medical intervention (Pedrizzetti et al., 2016; S2, Supplementary Methods).

The first step in calculating HDFs was to measure the diameters of both mitral and aortic valves (Supplementary Figures S2, S3, Supplementary Materials). Once the diameters of the mitral and aortic orifices were calculated, the temporal profile of the HDFs was used to obtain the following characteristic parameters of various phases of the cardiac cycle:

i. Left Ventricle Longitudinal Force (LVLF): Represents the average magnitude of the longitudinal force throughout the entire cardiac cycle; since it includes both positive and negative values, the magnitude was calculated as the root mean square of all values.
ii. Left Ventricle Systolic Longitudinal Force (LVsysLF): Calculated similarly to LVLF but confined exclusively to the systolic phase.
iii. Left Ventricle Impulse (LVim): Represents the average longitudinal force during the propulsive systolic phase, when the force is positive (directed from the LV towards the aorta); it is the area under the curve of the positive force profile during systole, normalized to the corresponding time interval.
iv. Left Ventricle Suction (LVs): Represents the average longitudinal force during the period following propulsion, when the force is negative.

HDFs will be used in the analysis of results to integrate information about volume and deformation with data regarding cardiac fluid dynamics.

#### 2.3.2 Left atrium

**FIGURE 2:**
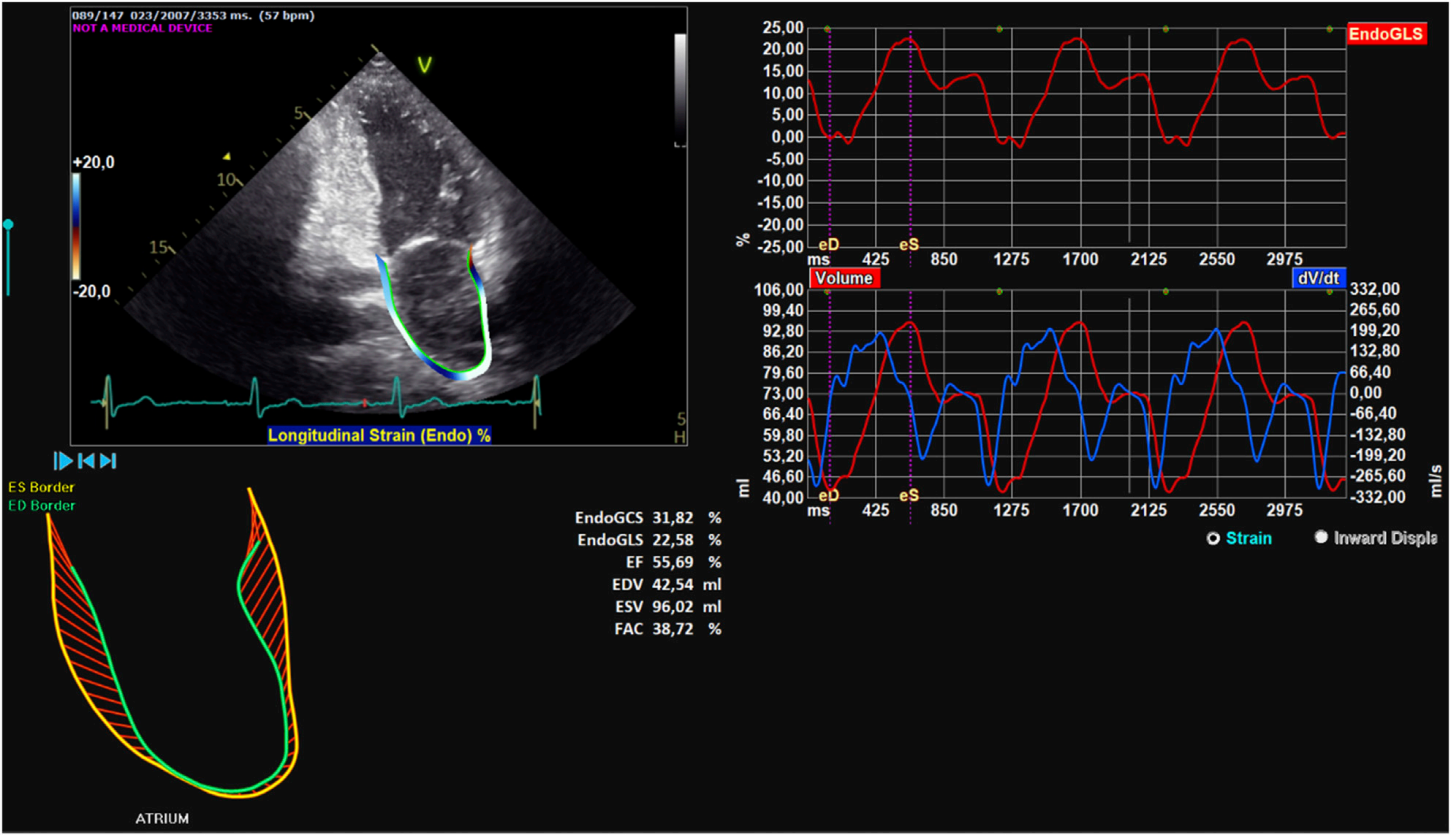
Left atrial strain. Global analysis of a patient’s LA with endocardial contour and retrieval of ENDO GCS, ENDO GLS, EDV, ESV, and EF parameters. On the top left, the endocardial contour of the LA. On the bottom left, LA end-systolic and end-diastolic contours, shown in yellow and green, respectively. On the top right, the strain trend throughout the entire heartbeat. On the bottom right, the volumetric trend throughout the entire heartbeat (in red) and its temporal derivative (in blue).

Unlike the approach used for LV image analysis, the analysis of LA 2D strain was conducted using only the two-chamber apical view (Figure 2). In this case as well, we manually traced the LA end-systolic borders, followed by the adjustment of the borders at end-diastole, throughout the entire cardiac cycle without altering the previously drawn contours. This approach allowed for the calculation of ENDO GCS, ENDO GLS, ESV, and EDV, as well as the measurement of atrial ejection fraction (LAEF), defined as that force exerted by the LA to accelerate the blood into the LV during atrial systole. The software performs detailed strain and volume parameters measurements for the LA, clearly outlining a difference from the number of parameters previously assessed for the LV. The endocardial contours at the end of systole (yellow) and the end of diastole (green) are reversed. This is because during systole the atrium fills and is at its maximum volume (while the ventricle contracts and its volume is at its minimum); during diastole, the atrium contracts and is at its minimum volume (while the ventricle in this phase fills and its volume is at its maximum). While the LV is subject to more extensive measurements, for the LA, the acquisition is limited to deformation (strain) and volume parameters, without extending the analysis to HDFs. This choice is motivated by the distinct, albeit interconnected, functionality of the LA and the LV in cardiac dynamics. The atrium, with a simpler anatomical structure compared to the ventricle and essentially predisposed to receiving and transferring blood to the ventricles, does not require the same depth of hemodynamic analysis as the ventricle, whose primary role is to pump blood throughout the entire circulatory system. In this context, measurements focused on atrial strain and volume emerge as sufficient metrics to assess atrial contractile function and filling. On the other hand, the joint analysis of these parameters for both LA and LV contribute to a thorough and comprehensive assessment of overall cardiac dynamics.

### 2.4 Hybrid atrial fibrillation ablation procedure

Hybrid AF ablation procedure has been previously described in detail by our group (De Asmundis et al., 2017). A transthoracic echocardiogram (TTE) was performed within 1 week prior to the procedure to assess LV function and exclude significant structural and/or valvular disease. On the day of the procedure, transoesophageal echocardiography (TOE) was performed to exclude intracardiac thrombus and pulmonary function testing was also performed as pre-procedure routine. The hybrid AF ablation was performed during a one-step procedure: (i) thoracoscopic ablation followed by (ii) endocardial mapping and eventual ablation. The procedure was performed in the hybrid electrophysiology laboratory as previously described (De Asmundis et al., 2017). If hybrid AF ablation was performed as first (index) procedure, antral PVI was performed with four to six applications using a bipolar radiofrequency (RF) clamp (Atricure, Inc., West Chester, OH, USA). After clamping, PVI was assessed by epicardial pacing through a quadripolar catheter (exit block). LAPWI was performed epicardially in all patients with two lines: (i) a roof line (connecting both superior PVs) and (ii) inferior line (connecting both inferior PVs). A bipolar RF pen or a linear pen device (Isolator Pen and Coolrail, Atricure Inc.) was used for LAPWI. LAA clipping was performed in all patients with the AtriClip device (AtriCure Inc.). After epicardial ablation all patients underwent to endocardial remap and eventual ablation during the same procedure.

### 2.5 Follow-up

After discharge, patients were scheduled for follow-up visits with baseline electrocardiogram (ECG) and 24 h Holter recordings at 3, 6, 12 months, and every 6 months after 1 year. Furthermore, a 7-day ECG Holter monitoring was recorded at 3, 6, and 12 months for the first year and then every 6 months. Primary endpoint was recurrence of any atrial tachyarrhythmias (ATas) defined as episodes >30 s after a 90-day post-ablation blanking period (BP) off antiarrhythmic drugs (AADs). Recurrence was assessed with standard ECG or 24 h ECG Holter monitoring or with implantable loop recorders or implanted devices interrogation if applicable. Moreover, a 24 h Holter monitoring was performed if any symptom following ablation was deemed as prompting further clinical investigation. Complications were adjudicated and analysed following current AF guidelines (Hindricks et al., 2021).

### 2.6 Statistical analysis

The analysis was conducted using JASP software, version 0.18.1 (University of Amsterdam, Amsterdam, Holland). All variables were tested for normality using the Shapiro-Wilk test. Normally distributed variables were described as mean ± standard deviation, and groups were compared using ANOVA to assess differences across the three time points (pre, post, and follow-up). Paired Student's t-tests were used for pairwise comparisons of the time points (pre-post, pre-follow-up, and post-follow-up). Variables not normally distributed were described as median (interquartile range), and comparisons across the three time points were made using the Friedman test, while pairwise comparisons were conducted using the Wilcoxon signed-rank test.

The parallel coordinates plots were created in MATLAB (MathWorks, Natick, MA, USA) to visualize the variation among the medians of LA and LV mechanics parameters across three time points in relation to the ablation.

A *P* value of less than 0.05 was considered statistically significant.

## 3 Results

### 3.1 Study population characteristics

A total of 50 patients were included in the study (70% males and 30% females, with an average age of 72 ± 9.0 years, age range 58–86). The clinical data for the entire cohort are presented in Table 1. All patients underwent to hybrid AF ablation with PVI + LAPWI and surgical LAA closure for PersAF. All patients were in AF 1 week before ablation. Baseline LVEF was 46.3 ± 13.8 mL (21.4), LAEF was 40 ± 14.7 mL and LAEDV was 53.8 ± 31 mL. Total of 31 patients (62%) were on AADs.

**TABLE 1:** Clinical characteristics of hybrid atrial fibrillation ablation patients.

	Hybrid ablation + LAA closure (*N = 50*)
Age at ablation (years)	72 ± 9.0
Gender (male), n (%)	35 (70.0)
BMI (kg/m2)	28.7 ± 4.6
Race/ethnicity, n (%)	
White/Caucasian, n (%)	49 (98.0)
Black/African, n (%)	1 (2.0)
Asian, n (%)	0 (0.8)
PersAF, n (%)	50 (100.0)
Dyslipidaemia, n (%)	11 (22.0)
Heart failure, n (%)	5 (10.0)
Hypertension, n (%)	25 (50.0)
Diabetes, n (%)	6 (12.0)
Stroke history, n (%)	3 (6.0)
Peripheral vascular disease, n (%)	2 (4.0)
CHA2DS2-VASc score	2.2 ± 5.8
LVEF (%)	46.3 ± 13.8
LVEF (%)	45 (21.4)
LAEF (%)	40 ± 14.7
LAVI (mL/m2)	53.8 ± 31
AADs, n (%)	31 (62.0)
Flecainide, n (%)	8 (16.0)
Propafenone, n (%)	1 (2.0)
Beta-blockers, n (%)	19 (38.0)
Sotalol, n (%)	10 (20.0)
Amiodarone, n (%)	6 (12.0)
Calcium channel blockers, n (%)	1 (2.0)
VKA, n (%)	3 (6.0)
DOAC, n (%)	47 (94.0)

AADs, antiarrhythmic drugs; AF, atrial fibrillation; BMI, body mass index; DOAC, direct oral anticoagulant; LAVI, left atrium volume index; LVEF, left ventricular ejection fraction; LAEF, left atrial ejection fraction; VKA, vitamin K antagonists.

### 3.2 Ventricular mechanics variations

There was an increase in LV ENDO GCS post-ablation compared to baseline [LV ENDO GCS post-ablation −26.7% ± 5.7% vs LV ENDO GCS baseline −22.5% ± 8%, *P* < 0.001] and there was no change in LV ENDO GCS between post-ablation and follow-up [LV ENDO GCS post-ablation −26.7% ± 5.7% vs LA ENDO GCS follow-up −26% ± 10.4%, *P* = 0.61]. Furthermore, LV ENDO GLS increased at post-ablation compared to baseline [LV ENDO GLS post-ablation −20.5% ± 5.7% vs LV ENDO GLS baseline −16.6% ± 7.3%, *P* < 0.001] and did not change between post-ablation and follow-up [LV ENDO GLS post-ablation −20.5% ± 5.7% vs LV ENDO GLS follow-up −19.6% ± 5.4%, *P* = 0.26]. LVEF post-ablation increased compared to baseline [LVEF post-ablation 54.6% ± 9.4% vs LVEF baseline 46.3% ± 13.8%, *P* < 0.001] and there was no change in LVEF between post-ablation and follow-up [LVEF post-ablation 54.6% ± 9.4% vs LVEF follow-up 54.3% ± 10.4%, *P* = 0.82]. LVEDV did not change after ablation compared to baseline [LVEDV post-ablation 118.4 ± 36.2 vs LVEDV baseline 118.9 ± 43, *P* = 0.87] and there was an increase between post-ablation and follow-up [LVEDV post-ablation 118.4 ± 36.2 vs LVEDV follow-up 138.3 ± 46.2, *P* < 0.001]. On the other hand, LVESV post-ablation decreased compared to baseline [LVESV post-ablation 53.3 ± 20.3 mL vs LVESV baseline 61.3 ± 23.5 mL, *P* = 0.001].

**FIGURE 3:**
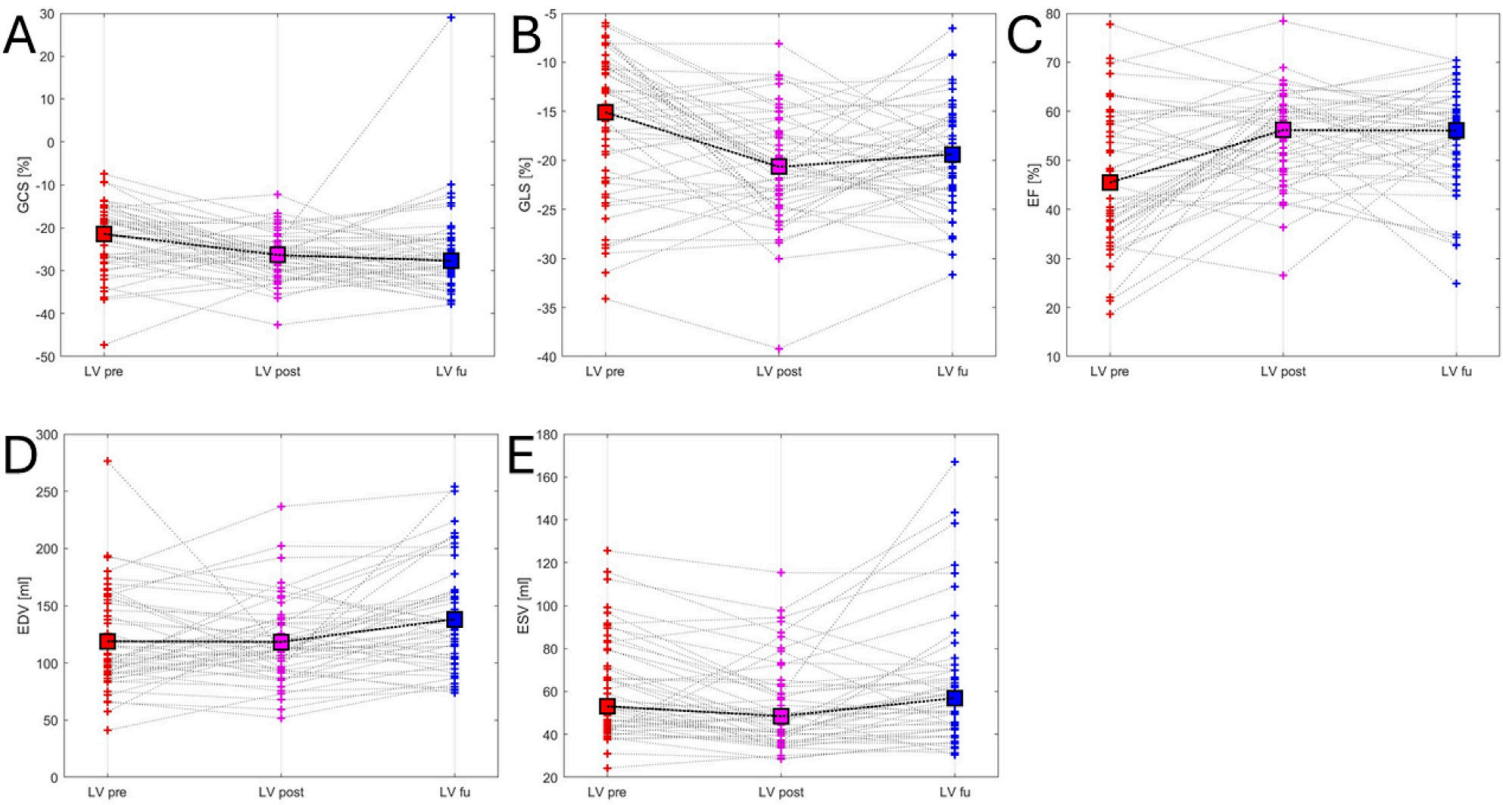
Left ventricular mechanics variations. This figure illustrates variations in left ventricular mechanics across the three time points: red (before ablation), purple (after ablation) and blue (follow-up). Panel **(A)** LV ENDO GCS - left ventricular global endocardial circumferential strain trend; Panel **(B)** LV ENDO GLS - left ventricular global endocardial longitudinal strain trend; Panel **(C)** LVEF - left ventricular ejection fraction trend; Panel **(D)** LVEDV - left ventricular end-diastolic volume trend; Panel **(E)** LVESV - left ventricular end-systolic volume trend.

**FIGURE 4:**
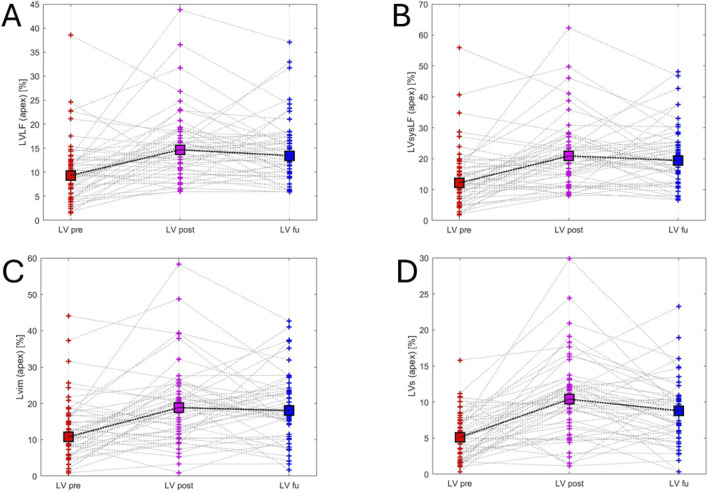
Left ventricular hemodynamic forces variations. This figure illustrates variations in left ventricular hemodynamic forces across the three time points: red (before ablation), purple (after ablation) and blue (follow-up). Panel **(A)** LVLF - left ventricular longitudinal force trend; Panel **(B)** LVsysLF - left ventricular systolic longitudinal force trend; Panel **(C)** LVim - left ventricular impulse trend; Panel **(D)** LVs left ventricular suction trend.

The study of ventricular mechanics was further conducted by analysing LV HDFs, and there was a significant increase in all forces immediately after the ablation. In particular, LVLF post-ablation increased compared to baseline [LVLF post-ablation 15.5% ± 7.5% vs LVLF baseline 10.4% ± 6.7%, *P* < 0.001] and there was no change in LVLF between post-ablation and follow-up [LVLF post ablation 15.5% ± 7.5% vs LVLF follow-up 14.7% ± 7%, *P* = 0.34]. Furthermore, LVsysLF post-ablation increased compared to baseline [LVsysLF post-ablation 21.5% ± 11.3% vs LVsysLF baseline 14.1% ± 11%, *P* < 0.001] and there was no change in LVsysLF between post-ablation and follow-up [LVsysLF post-ablation 21.5% ± 11.3% vs LVsysLF follow-up 20.9% ± 10%, *P* = 0.67]. LVim also increased after the ablation [LVim post-ablation 19.6% ± 11.1% vs LVim baseline 12.8% ± 8.7%, *P* < 0.001] and there was no change in LVim between post-ablation and follow-up [LVim post-ablation 19.6% ± 11.1% vs LVim follow-up 19.2% ± 9.5%, *P* = 0.80]. Finally, there was an increase in LVs post-ablation compared to baseline [LVs post-ablation 10.6% ± 5.8% vs LVs baseline 5.4% ± 3.3%, *P* < 0.001] and at follow-up compared to baseline [LVs follow-up 8.7% ± 4.6% vs LVs baseline 5.4% ± 3.3%, *P* < 0.001] (Table 2; Supplementary Tables S1–S3; Figures 3, 4).

**TABLE 2:** Left ventricular cardiac mechanics variations in hybrid atrial fibrillation ablated patients.

	Baseline (*N = 50*)	Post- ablation (*N = 50*)	Follow-up (*N = 50*)	*P* Value
LV ENDO GCS (%)	−22.5 ± 8	−26.7 ± 5.7	−26 ± 10.4	0.001
LV ENDO GLS (%)	−16.6 ± 7.3	−20.5 ± 5.7	−19.6 ± 5.4	0.050
LVEF (%)	46.3 ± 13.8	54.6 ± 9.4	54.3 ± 10.4	0.005
LVEDV (mL)	118.9 ± 43	118.4 ± 36.2	138.3 ± 46.2	<0.001
LVESV (mL)	61.3 ± 23.5	53.3 ± 20.3	63.5 ± 30.2	<0.001
LVLF (%)	10.4 ± 6.7	15.5 ± 7.5	14.7 ± 7	<0.001
LVsysLF (%)	14.1 ± 11	21.5 ± 11.3	20.9 ± 10	<0.001
LVim (%)	12.8 ± 8.7	19.6 ± 11.1	19.2 ± 9.5	0.006
LVs (%)	5.4 ± 3.3	10.6 ± 5.8	8.7 ± 4.6	<0.001

LV ENDO GCS, left ventricular global endocardial circumferential strain; LV ENDO GLS, left ventricular global endocardial longitudinal strain; LVEF, left ventricular ejection fraction; LVEDV, left ventricular end-diastolic volume; LVESV, left ventricular end-systolic volume; LVLF, left ventricular longitudinal force; LVsysLF, left ventricular systolic longitudinal force; LVim, left ventricular impulse; LVs, left ventricular suction.

### 3.3 Atrial mechanics variations

**FIGURE 5:**
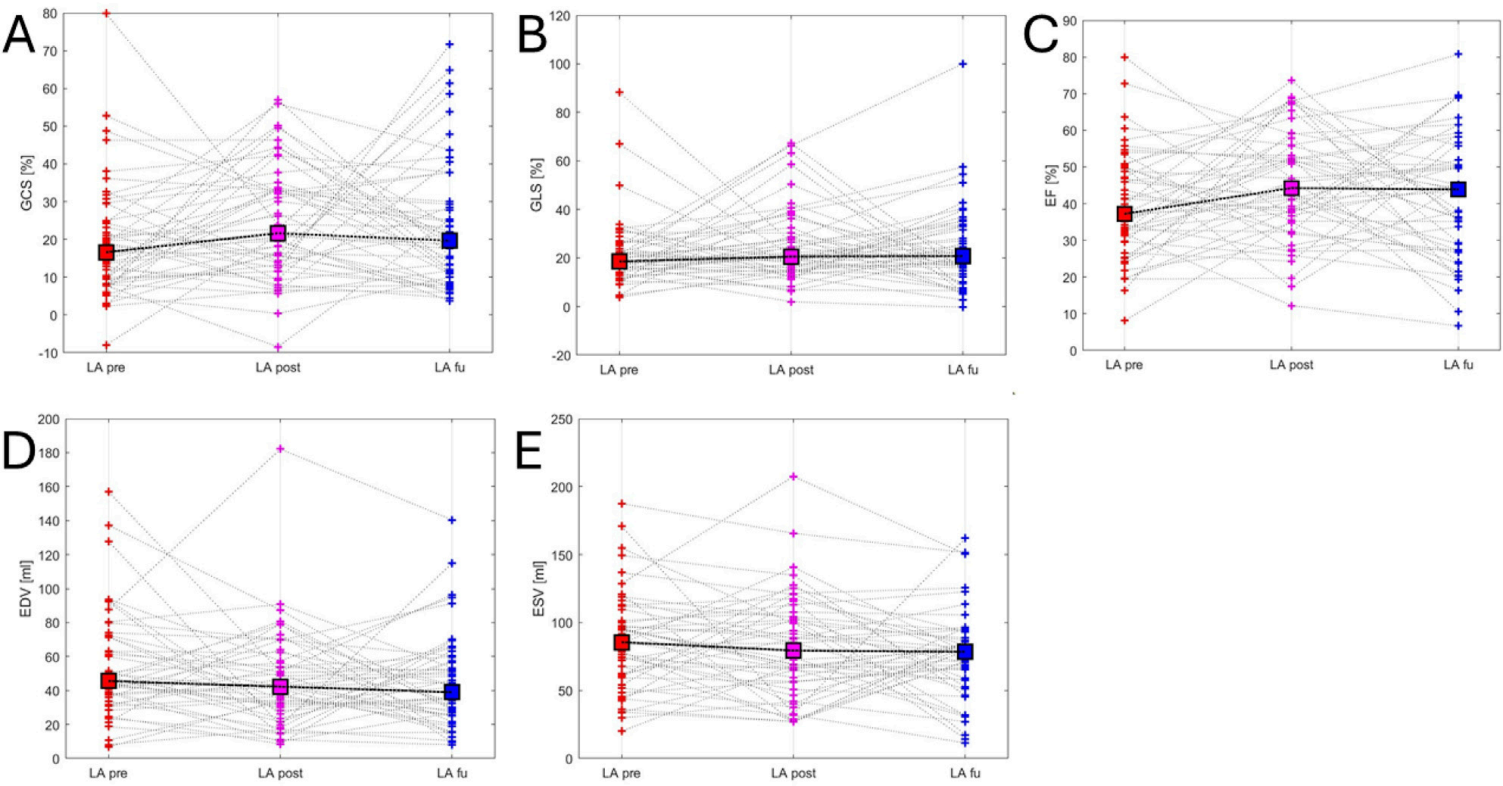
Left atrial mechanics variations. This figure illustrates variations in left atrial mechanics across the three time points: red (before ablation), purple (after ablation) and blue (follow-up). Panel **(A)** LA ENDO GCS - left atrial global endocardial circumferential strain trend; Panel **(B)** LA ENDO GLS - left atrial global endocardial longitudinal strain trend; Panel **(C)** LAEF - left atrial ejection fraction trend; Panel **(D)** LAEDV - left atrial end-diastolic volume trend; Panel **(E)** LAESV - left atrial end-systolic volume trend.

At follow-up, LAEDV decreased compared to LAEDV baseline [LAEDV follow-up 44.7 ± 27.61 mL vs LAEDV baseline 53.8 ± 31 mL, *P* = 0.025] and there was no change in LAEDV between baseline and post-ablation [LAEDV baseline 53.8 ± 31 mL vs LAEDV post-ablation 46.1 ± 29.1 mL, *P* = 0.13]. There was an increase in LA ENDO GCS post-ablation compared to baseline [LA ENDO GCS post-ablation 24.5% ± 15% vs LA ENDO GCS baseline 19.2% ± 15.2%, *P* = 0.042] and there was no change in LA ENDO GCS between post-ablation and follow-up [LA ENDO GCS post-ablation 24.5% ± 15% vs LA ENDO GCS follow-up 23.9% ± 17.1%, *P* = 0.3]. There was a significant change in LA ENDO GLS between baseline, post-ablation and follow-up [LA ENDO GLS baseline 21.5% ± 14.5% vs LA ENDO GLS post-ablation 25.2% ± 15.3% vs LA ENDO GLS follow-up 23.7 ± 17.3, *P* = 0.019]. There was no change in LAEF between baseline, post-ablation, and follow-up [LAEF baseline 40% ± 14.7% vs LAEF post-ablation 45% ± 14.8% vs LAEF follow-up 42.3% ± 17.2%, *P* = 0.14] and in LAESV between baseline, post-ablation and follow-up [LAESV baseline 86 ± 36.5 mL vs LAESV post-ablation 82.2 ± 38.8 mL vs LAESV follow-up 76.1 ± 32.6 mL, *P* = 0.2] (Table 3; Supplementary Tables S4–S6; Figure 5).

**TABLE 3:** Left atrial cardiac mechanics parameters variations in hybrid atrial fibrillation ablated patients.

	Baseline (N = 50)	Post-ablation (*N = 50*)	Follow-up (*N = 50*)	*P* Value
LA ENDO GCS (%)	19.2 ± 15.2	24.5 ± 15	23.9 ± 17.1	0.023
LA ENDO GLS (%)	21.5 ± 14.5	25.2 ± 15.3	23.7 ± 17.3	0.38
LAEF (%)	40 ± 14.7	45 ± 14.8	42.3 ± 17.2	0.14
LAEDV (mL)	53.8 ± 31	46.1 ± 29.1	44.7 ± 27.6	0.006
LAESV (mL)	86 ± 36.5	82.2 ± 38.8	76.1 ± 32.6	0.20

LA ENDO GCS, left atrial global endocardial circumferential strain; LA ENDO GLS, left atrial global endocardial longitudinal strain; LAEF, left atrial ejection fraction; LAEDV, left atrial end-diastolic volume; LAESV, left atrial end-systolic volume.

### 3.4 Follow-up

One week after ablation all patients were in sinus rhythm (SR) and at median follow-up of 62.3 months ±20.3, total of 34 patients (68%) were free from ATAs. Recurrence was observed in total of 16 patients (32%). There were no differences in both LA and LV mechanics parameters between patients with and without recurrences (Supplementary Tables S7, S8).

## 4 Discussion

The main results of this study can be summarized as follows: 1) After hybrid AF ablation with PVI + LAPWI and surgical LAA closure, there was a significant improvement in LVEF, LV strain and LV HDFs that was persistent at long-term follow-up; 2) After hybrid AF ablation with PVI + LAPWI and surgical LAA closure, there was a significant improvement in LA strain that was persistent at long-term follow-up; 3) Furthermore, there was a significant LA remodelling with a reduction of LAEDV after ablation + LAA closure that was consistent at follow-up.

### 4.1 Effects of hybrid atrial fibrillation ablation on left ventricular mechanics

The hemodynamic changes induced by AF continues to be a subject of study aiming at improving the clinical symptoms of patients affected by this arrhythmic pathology. Previous studies demonstrated that induced AF was associated with lower systolic blood pressure (Almroth et al., 2023). PersAF is associated with cardiac dysfunction, secondary to 1) mechanoelectrical feedback; 2) Neurohumoral Modulation; 3) Atrial Ionic Channel Remodeling (Cha et al., 2004). Building upon these evidences, randomized clinical trials demonstrated a significant improvement in outcomes after AF catheter ablation in patients with systolic dysfunction. Both the CASTLE-AF, in patients with LVEF <35% (Marrouche et al., 2018) and the CASTLE‐HTx trial (Sohns et al., 2023) in patients waiting for heart transplant, demonstrated higher survival in patients undergoing AF ablation compared to controls. In particular, in the CASTLE-AF, improvement in LVEF since baseline visit was higher in the ablation group. The mean increase in LVEF for patients ablated for PAF vs PersAF was 5.0% vs 8.7%.

The result of the current study on catheter ablation of patients with PersAF is consistent with previous results (Marrouche et al., 2018; Sohns et al., 2023), showing an increase in LVEF of 7%–8%. The results of our study highlight also an improvement in LV strain parameters. This is consistent with previous data from Tops et al., demonstrating that after successful catheter ablation, LV circumferential and longitudinal strain and strain rate significantly improved (Tops et al., 2009). A shortcoming of this study is the lack of post-ablation echocardiographic assessment; thus, the timing of improvement is unclear.

Our study is the first to demonstrate that these changes happen right after hybrid ablation, and they are persistent at follow-up.

### 4.2 Left ventricular hemodynamic forces after hybrid ablation

The current study is the first to perform an analysis of HDFs after AF ablation. Our results show an improvement in HDFs in the LV after hybrid ablation that was consistent at follow-up. Our findings demonstrate a significant increase in longitudinal forces (LVLF, LVsysLF, Lvim and LVs) following AF ablation, which may indicate improved synchronization and efficiency of LV contraction. This enhancement in longitudinal forces can be interpreted as a reflection of restored myocardial contractile function and alignment of myocardial fibres post-ablation, potentially leading to improved cardiac output and reduced workload on the myocardium. Such improvements may correlate with clinical outcomes such as reduced symptoms of heart failure, improved exercise tolerance, and potentially better long-term prognosis. Although further studies are needed to fully establish these parameters in routine clinical decision-making, the observed increase in longitudinal forces provides promising mechanistic insights into the benefits of ablation therapy. This can contribute, for example, to a reduction in the risk of heart failure and may explain the improvement in prognosis for patients with heart failure after AF ablation. The improvement of all these parameters occurs early right after the ablation and this improvement is persistent at follow-up. This demonstrates that the beneficial effect is not transient, further reinforcing the role of ablation and the prognostic advantage that persists even at long term follow-up.

### 4.3 Left atrial remodelling after hybrid ablation

The results of this study show an improvement in LA volume and function after ablation + LAA closure that is persistent at follow-up. Reduction in LA diameter (mean reduction, 3.6–4.7 mm) was observed after AF ablation in the CASTLE-AF. However, LAEDV has shown better correlation with AF risk (Njoku et al., 2018). In the study by Liu et al. (2022), the authors observed a significant reduction of LAEDV after AF catheter ablation, but only in patients with PersAF. Our results show a reduction of LAEDV of 17%, that is higher compared with 13.6% found by Liu et al. This might be explained by the additional LAA clipping performed in our study. In previous research from our group (Marini et al., 2022b), on stand-alone LAA epicardial clipping (no ablation), at 2D strain of LA, the reservoir function decreased significantly at discharge, compared to baseline, and recovered at 3-month follow-up. Adding ablation to LAA clip (PVI + LAPWI) in the current study determined an improvement in LA strain after ablation that was maintained at follow-up. This is consistent with the study by Liu et al. showing in the PersAF an improvement of LA strain within 1 week after AF ablation and a further gradual increase at follow-up. Further delving into the variations observed in LA, the reduction in its volumes can be explained in several ways. Maintaining a stable SR results in electrical remodelling of the atrium (Cha et al., 2004), which is reflected in mechanical remodelling, leading to a reduction in atrial volumes. In addition, the effect of closing the LAA contributes to a mechanical volumetric reduction. This may result in a secondary volumetric reduction associated with a neuro-hormonal alteration related to the production of BNP (Marini et al., 2022b). Also, LAEF increases due to the reduction in end-diastolic and end-systolic volumes. This can be explained on the one hand by volumetric remodelling of the LA, but on the other hand, by the effect of ablation and thus the elimination of a mechanical component of part of the LA. Moreover, there is an improvement in atrial strain immediately after ablation and at follow-up. This might be secondary to the maintenance of a stable SR and therefore better shortening of the remaining atrial segments (not ablated), particularly the anterior wall (Cha et al., 2004).

## 5 Limitations

This study, being retrospective in nature, has certain limitations that affect its applicability and the generalizability of the results. First, it should be noted that the study was conducted at a single centre specializing in hybrid AF ablation. The relatively small sample size, composed primarily of individuals of Caucasian ethnicity, limits the generalizability of the findings to the broader population. However, it is important to recognize that the rigorous selection of subjects and the in-depth image analysis involved considerable effort, still providing a reasonable sample within the context of a single-centre study. Second, while the addition of LAA closure in all patients represents a unique approach, the absence of a control group limits our ability to discern whether the LAA closure itself contributes independently to the observed improvements in LA volumes and strain. In future studies, including a control group will be essential to isolate the effects of LAA closure. Another limitation of this study is the absence of a comparison group, such as patients who underwent standalone ablation. We did not include patients who underwent hybrid AF ablation without LAA closure due to the invasiveness of the procedure and the complexity of the patients. For this reason, in our centre we prefer to perform a single-step approach that includes both ablation and LAA closure. While the absence of a comparison group may limit the ability to distinguish the effects of each procedure, several studies have shown that standalone catheter ablation can lead to improvements in LA mechanics (Xiong et al., 2015) and LVEF (Sugumar et al., 2019), while standalone LAA occlusion may not yield similar improvements (Marini et al., 2022c). These limitations are intentional, as our manuscript specifically focuses on evaluating the hybrid procedure combined with LAA surgical, which we believe provides a novel and important contribution to understanding the mechanical and hemodynamic effects of this approach in patients with AF. Future studies comparing these approaches with standalone ablation or LAA occlusion will help address this gap. Third, we recognize that strain parameters are likely to differ in patients with SR compared to those with AF. Thus, it remains unclear whether the observed results are more attributable to the restoration of SR or specifically to the ablation technique. Next study will aim to include a cohort of cardioverted patients to address this distinction. Additionally, we did not validate the strain measurements against reference standards, such as cardiac magnetic resonance (CMR), which may limit the accuracy of our strain analysis. We also acknowledge that strain measurements may vary between different ultrasound vendors due to differences in analytical algorithms, potentially introducing intervendor variability. The apical approach for GCS can also be challenging, as it may be less precise due to ultrasound’s limited lateral resolution or incomplete circumferential visualization from apical views. Although prior studies have shown that lateral resolution is not a critical limitation (Pedrizzetti et al., 2019), we used triplane evaluation to reduce artifacts in deformation that may arise from out-of-plane displacement in a 3D structure. Lastly, our estimations of HDFs depend on 2D image quality and frame rates, which should be considered when interpreting these findings.

## 6 Conclusion

Hybrid AF ablation + LAA clipping in patients with PersAF is associated with improvements in both LV and LA mechanics, including HDFs, LVEF, ventricular strain and atrial strain. Furthermore, significant LA remodelling was observed, with a reduction of LAEDV. These changes occur early after the procedure and are persistent at follow-up. Further studies are needed to assess the value of the different procedural steps on the observed results (PVI + LAPWI vs. LAA epicardial clipping).

## Data Availability

The raw data supporting the conclusions of this article will be made available by the authors, without undue reservation.
