# Supplementary material for: 2D speckle-tracking echocardiography assessment of left atrial and left ventricular mechanics: outcomes in patients with atrial fibrillation treated with hybrid ablation and left atrial appendage surgical closure
Source: Front Bioeng Biotechnol. 2025 Mar 12;13:1538809. doi: 10.3389/fbioe.2025.1538809 (PMC11937039; doi:10.3389/fbioe.2025.1538809)
Supplement: Supplementary file 1 [file DataSheet1.docx]

SUPPLEMENTARY MATERIALS

SUPPLEMENTARY METHODS

S1. Strain

ENDO GCS [%] represents the change in length (shortening during systole, represented as a negative strain value) of the endocardium along the circumferential axis of the left ventricle, while ENDO GLS [%] represents an analysis of endocardial strain (shortening during systole, represented as a negative strain value) that primarily reflects the function of the longitudinally oriented sub-endocardial fibres, which are most susceptible to ischemic damage and wall stress. Essentially, GCS is an indicator of the heart's ability to contract and relax effectively in a circumferential direction, while GLS is an indicator of the heart's ability to contract and relax effectively in a longitudinal direction.

It is appropriate to define these parameters through a theoretical recall starting from the concept of *strain*. This approach aims to provide the reader with a solid conceptual foundation to fully understand these concepts. The attempt to track tissue movement from video frames is merely a starting point for measuring heart deformation. It is assumed that the points traced during the contour tracking represent distinct parts of the tissue that can move relative to specific spatial coordinates. These points can move radially (usually perpendicular to the local wall plane), longitudinally, or circumferentially along the heart wall, depending on whether the image is taken along or across the wall. The velocity vector of the tissue can be calculated as the displacement between one frame and the next divided by the elapsed time. However, since this involves temporal derivatives (calculated from the difference in displacement values at different times), it is more susceptible to noise, reduces precision, and requires a sufficiently high frame acquisition rate. Understanding the movement of multiple tissue regions allows us to calculate the tissue deformation between them. Deformation can be expressed either as a decimal fraction, for example, 0.15, or as a percentage, such as 15%. Expressing it as a percentage can lead to confusion and potential misunderstandings during reporting, as percentages may have two meanings: absolute differences in strain measurements or proportional differences between groups. Therefore, it is crucial to describe and interpret such results with great care. When discussing strain, it essentially records whether the length 𝐿 of a piece of tissue decreases (shortening or thinning, known as negative strain) or increases (lengthening or thickening, known as positive strain) relative to a diastolic initial length $L_{0}$​. Based on this, strain can be defined as:

$$St= \frac{L-L_{0}}{L_{0}}$$

*(1)*

This is known as *Lagrangian strain* because it refers to an undeformed initial state. In some applications, a different definition called *Eulerian strain* has been used, where the same difference in lengths is normalized by the final length $L$, instead of $L_{0}$, in the denominator of equation (1); however, the use of this formula is uncommon in cardiology.

Strain can be calculated by taking a segment of tissue along a specific direction. When this segment is taken in the longitudinal direction, we obtain longitudinal strain; if taken along the circumference, we achieve circumferential strain; if measured across the thickness, we obtain radial strain. Endocardial longitudinal and circumferential strains are calculated when the segment length $L$ of the tissue is taken from the endocardial border. Endocardial strain values are the most used in clinical studies because they better represent the functional purpose of myocardial contraction, reducing the endocardial surface area of the cavity to eject the systolic volume, and as such, they correlate better with volumetric measurements such as ejection fraction. Similarly, epicardial strain, which is typically lower than endocardial strain, can be assessed along the epicardial border, although this is rarely used. Descriptions of displacements or strain must be expressed relative to an initial reference state, usually considered the end of diastole moment. Conversely, the speed and strain rate should reflect an instantaneous activity independent of a starting point. From the values of Lagrangian strain, the *Lagrangian strain rate* can be calculated as its derivative with respect to time:

$$SR_{L}=\frac{dSt}{dt}=\frac{1}{L_{0}}\frac{dL}{dt}$$

*(2)*

This means that, like velocity, the strain rate is a differential quantity, and its assessment depends on sufficient temporal resolution. Its accuracy is lower compared to that of strain. However, observing equation (2), it is evident that the definition of the Lagrangian strain rate depends on a reference length $L_{0}$a dependency that becomes problematic when defining an instantaneous property. For this reason, the Lagrangian strain rate is not commonly used. Instead, a physically consistent definition of the strain rate is known as the *natural strain rate*

$$SR=\frac{1}{L}\frac{dL}{dt}$$

*(3)*

where the rate of shortening of a tissue length $L$ is measured relative to the actual length of the tissue, independent of its previous deformation history. Lagrangian strain (1) and natural strain rate (3) are the most used metrics in cardiology, typically without the use of the Lagrangian/natural suffix. In this case, the relationship between strain and strain rate becomes:

$$SR=\frac{1}{St+1}\frac{dSt}{dt}, St=exp\left\{ \int_{t_{0}}^{t} SRdt \right\}-1$$

*(4)*

This apparent complexity arises from the need to define strain in an intuitive way (1), while a natural definition of strain as the integral of the natural strain rate, commonly referred to as *natural strain*

$${St}_{N}=\int_{t_{o}}^{t} SRdt=\log\frac{L}{L_{0}},$$

*(5)*

would have ensured greater mathematical consistency. However, definition (5) is less intuitive and cannot be immediately compared with visual measurements, nor is it practical for clinical cardiology [16].

S2. HDFs calculation

In recent years it has been demonstrated that the analysis of flow forces (hemodynamic forces) can be conducted using a mathematical model based on the first principle of fluid dynamics. This model utilizes knowledge of the geometry of the left ventricle, endocardial velocities, and the areas of the aortic and mitral orifices, calculated by measuring the internal diameter of the valve rings; thus, there is no need to assess the blood velocity within the left ventricle [17]. The total hemodynamic force, $F\left( t \right)$, exchanged between the blood volume and the endocardium, can be calculated through the momentum balance within the volume of the left ventricle $V\left( t \right)$:

$$F\left( t \right)=\rho\int_{V\left( t \right)} \frac{\partial v}{\partial t}dV+\int_{S(t)} vv_{n}dS$$

*(6)*

where $S\left( t \right)$is the surface surrounding the volume, $\rho$ is the fluid density, and $v$ is the velocity vector, with the subscript "$n$" indicating the outward normal component. The second term on the right side of the preceding formula represents the momentum flow through the instantaneous boundary of the volume of the left ventricle $S\left( t \right)$. The first term represents the inertia of the blood, calculated as the rate of change of velocity within the volume of the left ventricle. This can also be reformulated as the rate of change of velocity along the boundaries of the volume, allowing the calculation of force based on information evaluated on the surface surrounding the volume of the left ventricle:

$$F\left( t \right)=\rho\int_{S\left( t \right)} x\frac{\partial v}{\partial t}dS+\rho\int_{S(t)} vv_{n}dS$$

*(7)*

The longitudinal component of the hemodynamic force is derived from the previous formula by introducing the longitudinal position with the position vector $x$ in the first term, and the longitudinal velocity with the velocity vector $v$ in the second term. The surface $S\left( t \right)$that bounds the volume of the left ventricle consists of a closed boundary, which includes the endocardial surface and the closed part of the base, and an open boundary, represented by the mitral and aortic valves during the diastole and systole phases. The terms of the formula can thus be calculated based on the velocity of the tissues at the endocardium of the left ventricle and at the base, and from the average velocity through the open valve, obtained by dividing the volume rate of the left ventricle by the area of the valve. The average blood velocity through the valve can be evaluated by volumetric changes using the principle of mass conservation. Therefore, the estimation of the hemodynamic flow index from equation (7) can be integrated into current imaging solutions that calculate volumetric velocities or are dedicated to strain imaging, and thus can be applied to standard images recorded during an echocardiographic examination.

SUPPLEMENTARY TABLES

**Table S1** Left ventricular mechanics parameters variations before and after hybrid ablation

|  | Baseline  (*N=50*) | Post-ablation (*N=50*) | *P* value |
| --- | --- | --- | --- |
| LV ENDO GCS (%)  LV ENDO GLS (%) | -22.5 ± 8  -16.6 ± 7.3 | -26.7 ± 5.7  -20.5 ± 5.7 | < .001  < .001 |
| LVEF (%) | 46.3 ±13.8 | 54.6 ± 9.4 | < .001 |
| LVEDV (ml) | 118.9 ± 43 | 118.4 ± 36.2 | 0.87 |
| LVESV (ml) | 61.3 ± 23.5 | 53.3 ± 20.3 | 0.001 |
| LVLF (%) | 10.4 ± 6.7 | 15.5 ± 7.5 | < .001 |
| LVsysLF (%) | 14.1 ± 11 | 21.5 ± 11.3 | < .001 |
| LVim (%) | 12.8 ± 8.7 | 19.6 ± 11.1 | < .001 |
| LVs (%) | 5.4 ± 3.3 | 10.6 ± 5.8 | < .001 |

LV ENDO GCS, left ventricular global endocardial circumferential strain; LV ENDO GLS, left ventricular global endocardial longitudinal strain; LVEF, left ventricular ejection fraction; LVEDV, left ventricular end-diastolic volume, LVESV, left ventricular end-systolic volume; LVLF, left ventricular longitudinal force; LVsysLF, left ventricular systolic longitudinal force; LVim, left ventricular impulse; LVs, left ventricular suction.

**Table S2** Left ventricular mechanics parameters variations before hybrid ablation and after follow-up

|  | Baseline  (*N=50*) | Follow-up  (*N=50*) | *P* value |
| --- | --- | --- | --- |
| LV ENDO GCS (%)  LV ENDO GLS (%) | -22.5 ± 8  -16.6 ± 7.3 | -26 ± 10.4  -19.6 ± 5.4 | 0.003  0.008 |
| LVEF (%) | 46.3 ±13.8 | 54.3 ± 10.4 | < .001 |
| LVEDV (ml) | 118.9 ± 43 | 138.3 ± 46.2 | < .001 |
| LVESV (ml) | 61.3 ± 23.5 | 63.5 ± 30.2 | 0.46 |
| LVLF (%) | 10.4 ± 6.7 | 14.7 ± 7 | < .001 |
| LVsysLF (%) | 14.1 ± 11 | 20.9 ± 10 | < .001 |
| LVim (%) | 12.8 ± 8.7 | 19.2 ± 9.5 | < .001 |
| LVs (%) | 5.4 ± 3.3 | 8.7 ± 4.6 | < .001 |

LV ENDO GCS, left ventricular global endocardial circumferential strain; LV ENDO GLS, left ventricular global endocardial longitudinal strain; LVEF, left ventricular ejection fraction; LVEDV, left ventricular end-diastolic volume, LVESV, left ventricular end-systolic volume; LVLF, left ventricular longitudinal force; LVsysLF, left ventricular systolic longitudinal force; LVim, left ventricular impulse; LVs, left ventricular suction.

**Table S3** Left ventricular mechanics parameters variations after hybrid ablation and after follow-up

|  | Post-ablation (*N=50*) | Follow-up  (*N=50*) | *P* value |
| --- | --- | --- | --- |
| LV ENDO GCS (%)  LV ENDO GLS (%) | -26.7 ± 5.7  -20.5 ± 5.7 | -26 ± 10.4  -19.6 ± 5.4 | 0.61  0.26 |
| LVEF (%) | 54.6 ± 9.4 | 54.3 ± 10.4 | 0.82 |
| LVEDV (ml) | 118.4 ± 36.2 | 138.3 ± 46.2 | < .001 |
| LVESV (ml) | 53.3 ± 20.3 | 63.5 ± 30.2 | < .001 |
| LVLF (%) | 15.5 ± 7.5 | 14.7 ± 7 | 0.34 |
| LVsysLF (%) | 21.5 ± 11.3 | 20.9 ± 10 | 0.67 |
| LVim (%) | 19.6 ± 11.1 | 19.2 ± 9.5 | 0.80 |
| LVs (%) | 10.6 ± 5.8 | 8.7 ± 4.6 | 0.012 |

LV ENDO GCS, left ventricular global endocardial circumferential strain; LV ENDO GLS, left ventricular global endocardial longitudinal strain; LVEF, left ventricular ejection fraction; LVEDV, left ventricular end-diastolic volume, LVESV, left ventricular end-systolic volume; LVLF, left ventricular longitudinal force; LVsysLF, left ventricular systolic longitudinal force; LVim, left ventricular impulse; LVs, left ventricular suction.

**Table S4** Left atrial mechanics parameters variations before and after hybrid ablation

|  | Baseline  (*N=50*) | Post-ablation (*N=50*) | *P* value |
| --- | --- | --- | --- |
| LA ENDO GCS (%)  LA ENDO GLS (%) | 19.2 ± 15.2  21.5 ± 14.5 | 24.5 ± 15  25.2 ± 15.3 | 0.042  0.16 |
| LAEF (%) | 40 ± 14.7 | 45 ± 14.8 | 0.044 |
| LAEDV (ml) | 53.8 ± 31 | 46.1 ± 29.1 | 0.13 |
| LAESV (ml) | 86 ± 36.5 | 82.2 ± 38.8 | 0.36 |

LA ENDO GCS, left atrial global endocardial circumferential strain; LA ENDO GLS, left atrial global endocardial longitudinal strain; LAEF, left atrial ejection fraction; LAEDV, left atrial end-diastolic volume, LAESV, left atrial end-systolic volume.

**Table S5** Left atrial mechanics parameters variations before hybrid ablation and after follow-up

|  | Baseline  (*N=50*) | Follow-up  (*N=50*) | *P* value |
| --- | --- | --- | --- |
| LA ENDO GCS (%)  LA ENDO GLS (%) | 19.2 ± 15.2  21.5 ± 14.5 | 23.9 ± 17.1  23.7 ± 17.3 | 0.12  0.39 |
| LAEF (%) | 40 ± 14.7 | 42.3 ± 17.2 | 0.29 |
| LAEDV (ml) | 53.8 ± 31 | 44.7 ± 27.6 | 0.025 |
| LAESV (ml) | 86 ± 36.5 | 76.1 ± 32.6 | 0.05 |

LA ENDO GCS, left atrial global endocardial circumferential strain; LA ENDO GLS, left atrial global endocardial longitudinal strain; LAEF, left atrial ejection fraction; LAEDV, left atrial end-diastolic volume, LAESV, left atrial end-systolic volume.

**Table S6** Left atrial mechanics parameters variations after hybrid ablation and after follow-up

|  | Post-ablation (*N=50*) | Follow-up  (*N=50*) | *P* value |
| --- | --- | --- | --- |
| LA ENDO GCS (%)  LA ENDO GLS (%) | 24.5 ± 15  25.2 ± 15.3 | 23.9 ± 17.1  23.7 ± 17.3 | 0.30  0.62 |
| LAEF (%) | 45 ± 14.8 | 42.3 ± 17.2 | 0.36 |
| LAEDV (ml) | 46.1 ± 29.1 | 44.7 ± 27.6 | 0.71 |
| LAESV (ml) | 82.2 ± 38.8 | 76.1 ± 32.6 | 0.26 |

LA ENDO GCS, left atrial global endocardial circumferential strain; LA ENDO GLS, left atrial global endocardial longitudinal strain; LAEF, left atrial ejection fraction; LAEDV, left atrial end-diastolic volume, LAESV, left atrial end-systolic volume.

**Table S7** Left atrial mechanics parameters variations between patients with and without recurrence at follow-up

|  | No recurrence (*N=34*) | Recurrence  (*N=16*) | *P* value |
| --- | --- | --- | --- |
| LA ENDO GCS (%)  LA ENDO GLS (%) | 23.1 ± 17  23.5 ± 19 | 25.7 ± 19  24.1 ± 14 | 0.63  0.91 |
| LAEF (%) | 42.3 ± 18 | 42.3 ± 16 | 0.99 |
| LAEDV (ml) | 44.7 ± 27 | 44.8 ± 29 | 0.86 |
| LAESV (ml) | 75.5 ± 29 | 77.5 ± 40 | 0.99 |

LA ENDO GCS, left atrial global endocardial circumferential strain; LA ENDO GLS, left atrial global endocardial longitudinal strain; LAEF, left atrial ejection fraction; LAEDV, left atrial end-diastolic volume, LAESV, left atrial end-systolic volume.

**Table S8** Left ventricular mechanics parameters variations between patients with and without recurrence at follow-up

|  | No recurrence (*N=34*) | Recurrence  (*N=16*) | *P* value |
| --- | --- | --- | --- |
| LV ENDO GCS (%)  LV ENDO GLS (%) | -26.0 ± 11  -19.5 ± 5 | -25.8 ± 7  -19.9 ± 4 | 0.76  0.78 |
| LVEF (%) | 55.0 ± 10 | 52.3 ± 10 | 0.48 |
| LVEDV (ml) | 134.1 ± 39 | 147.3 ± 58 | 0.57 |
| LVESV (ml) | 60.3 ± 25 | 70.5 ± 38 | 0.45 |
| LVLF (%) | 14.1 ± 5 | 16.1 ± 9 | 0.89 |
| LVsysLF (%) | 20.3 ± 7 | 22.2 ± 13 | 0.53 |
| LVim (%) | 18.6 ± 7 | 19.2 ± 12 | 0.84 |
| LVs (%) | 8.6 ± 3 | 8.7 ± 6.6 | 0.62 |

LV ENDO GCS, left ventricular global endocardial circumferential strain; LV ENDO GLS, left ventricular global endocardial longitudinal strain; LVEF, left ventricular ejection fraction; LVEDV, left ventricular end-diastolic volume, LVESV, left ventricular end-systolic volume; LVLF, left ventricular longitudinal force; LVsysLF, left ventricular systolic longitudinal force; LVim, left ventricular impulse; LVs, left ventricular suction.

SUPPLEMENTARY FIGURES


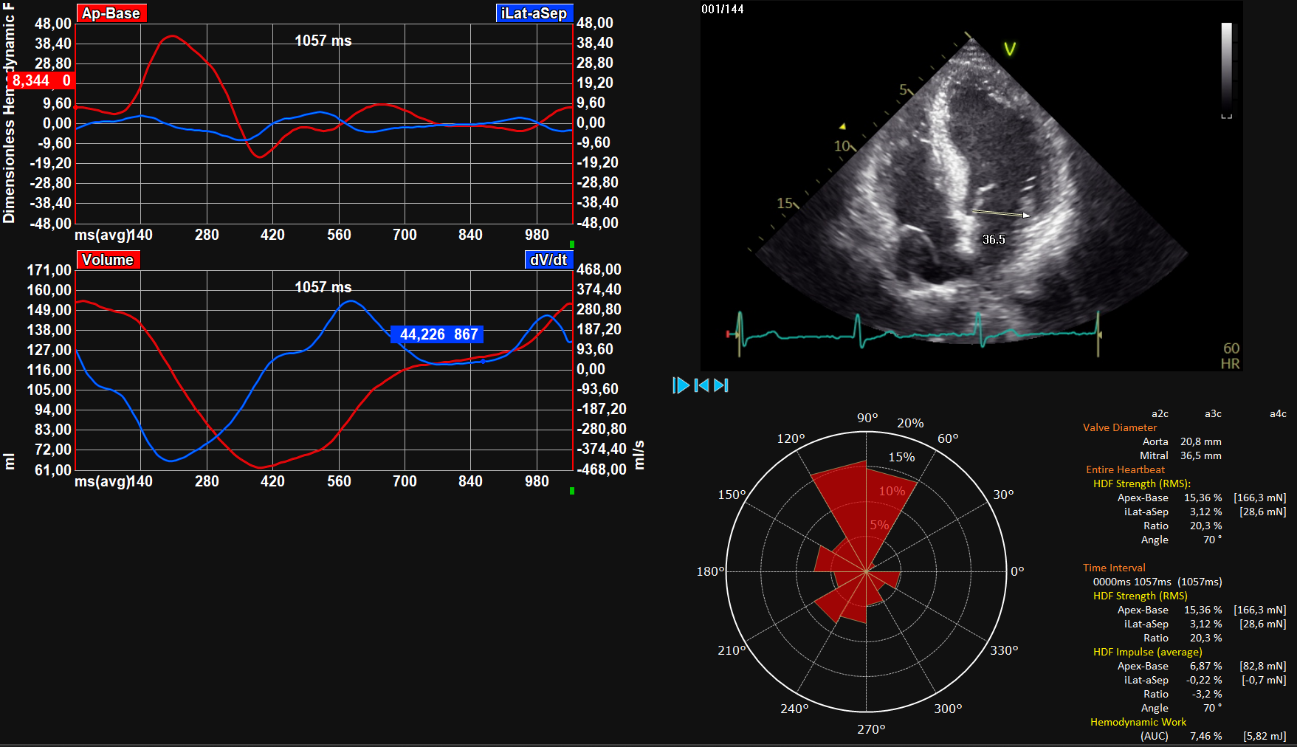
**Figure S1** Left ventricular HDFs calculation

LV HDFs calculation: On the top right, the strain trend throughout the entire heartbeat; on the bottom right, the volumetric trend during the entire heartbeat (in red) and its temporal derivative (in blue). On the top left, the four-chamber echo view with the mitral valve diameter calculation; on the bottom left, the HDFs bull’s-eye.

*LV HDFs, left ventricular hemodynamic forces.*

**Figure S2** Mitral valve diameter measurement


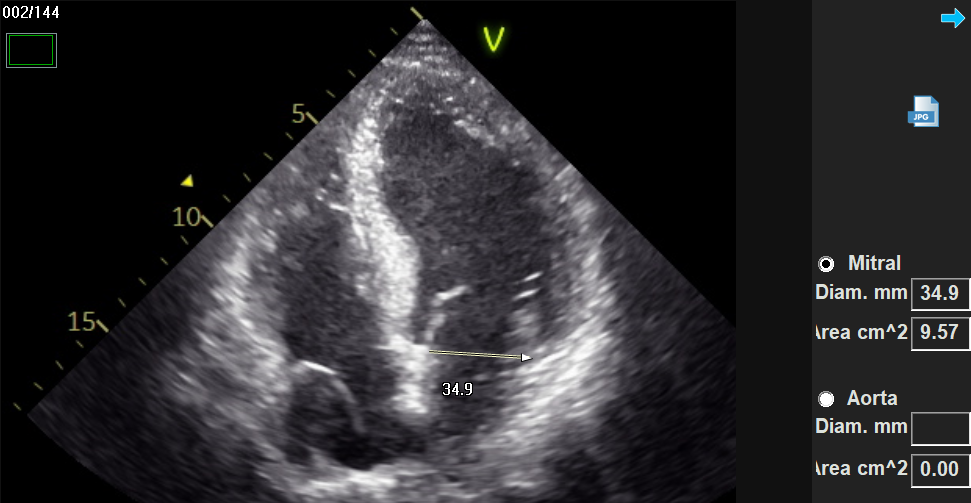
Measurement of the mitral valve diameter using a four-chambers apical view

**Figure S3** Aortic valve diameter measurement

**
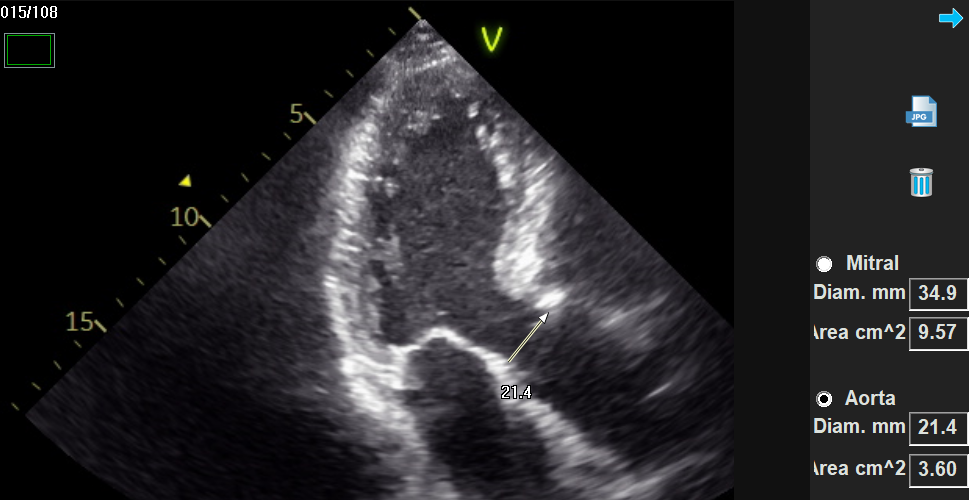
**

Measurement of the aortic valve diameter using a three-chambers apical view

*LA, left atrium; ENDO GCS, global endocardial circumferential strain; ENDO GLS, global endocardial longitudinal strain; EDV, end-diastolic volume; ESV, end-systolic volume; EF, ejection fraction.*
